# Coexistence of Microbial Species in Structured Communities by Forming a Hawk-Dove Game Like Interactive Relationship

**DOI:** 10.3389/fmicb.2019.00807

**Published:** 2019-04-16

**Authors:** Kelei Zhao, Jing Li, Ting Huang, Yang Yuan, Jiafu Lin, Bisong Yue, Xinrong Wang, Yiwen Chu

**Affiliations:** ^1^Antibiotics Research and Re-evaluation Key Laboratory of Sichuan Province, Sichuan Industrial Institute of Antibiotics, Chengdu University, Chengdu, China; ^2^Key Laboratory of Bio-Resources and Eco-Environment (Ministry of Education), College of Life Sciences, Sichuan University, Chengdu, China

**Keywords:** social microbiology, community, interspecific interaction, competition, coexistence, transcriptome

## Abstract

Microorganisms evolve kinds of elaborate interaction models that can form relatively stable communities in a wide range of ecosystems. It is recognized that the spatial genetic structure of microbes in surface-attached environments lays a good foundation for the persistence of polymicrobial communities in adverse conditions. However, the interacting dynamics of microbes in facilitating the formation and stabilization of community structure still remains elusive. In this study, we identify a hawk-dove game like interspecific relationship between the two Gram-negative opportunistic pathogens *Pseudomonas aeruginosa* and *Klebsiella pneumoniae*, which naturally coexist in insect gut and can cocolonize human tissues. Specifically, although *P. aeruginosa* had significant competitive advantage over cocultured *K. pneumoniae* on solid medium with rich nutrient factors, *K. pneumoniae* could resist the suppression of *P. aeruginosa* by enhancing the expression of membrane transporters induced by the extracellular metabolites of *P. aeruginosa*. By contrast, under the condition that *K. pneumoniae* had a growth advantage but *P. aeruginosa* met a metabolic burden in producing quorum-sensing-controlled extracellular products, the frequency of *K. pneumoniae* would be slightly higher than *P. aeruginosa* during the coexistence because *K. pneumoniae* was also capable of exploiting the extracellular metabolite from *P. aeruginosa*. In addition, *P. aeruginosa* quorum-sensing variant could reap benefits from *K. pneumoniae* in turn and reach a relatively stable two species equilibrium. These findings provide an explanation for the formation and maintenance of polymicrobial communities in different spatially structured environments, and thus may contribute to understanding the complex interspecific interactions of microbes in local communities and shed new light on the development of social microbiology.

## Introduction

Microorganisms are ubiquitous inhabitants of natural and unnatural ecosystems, and growth of them in a sessile lifestyle inevitably form communities composed of homo-/heterogeneous species with complex ecological interacting networks (Griffin et al., 2004; Adnani et al., 2017; Tshikantwa et al., 2018). Microbial species in symbiotic communities evolve kinds of chemical or physical interaction patterns such as metabolite-mediated cooperation/conflict and space competition, and these interactions can be beneficial, neutral or harmful to the fitness of individuals in the community. Accordingly, the outcomes of microbial interspecific interactions can be mutualistic, commensalistic or parasitic, by which may integrate the local species to adhere to material surfaces or persistently cocolonize host tissues (Xavier, 2011; Hussa and Goodrich-Blair, 2013; Short et al., 2014; Scherlach and Hertweck, 2018).

Surface-attached polymicrobial communities are frequently densely packed, and this leads to the formation of spatial genetic structure and promotes the evolution of cooperation in digesting macromolecular compound using extracellular enzymes (Chao and Levin, 1981; Greig and Travisano, 2003; Nadell et al., 2009; Elias and Banin, 2012; Frost et al., 2018; Solden et al., 2018). Nutrient limitation as a hallmark of dense communities can result in social competition for extracellular products that significantly benefit the producers and neighboring recipients. This creates selection for individuals with deficiency in producing the key factor but can reap the benefits of producers’ investment (Hansen et al., 2007; Xavier et al., 2011; Zengler and Zaramela, 2018). Theoretically, such kind of exploitive competition may occur in many aspects involving extracellular digestive proteases, antibiotic resistance enzymes and iron chelate, biofilm production, virulence determinants and so on (Diggle et al., 2007a; Brockhurst et al., 2008, 2010; Gore et al., 2009; Diard et al., 2013; van Gestel et al., 2014; Kelsic et al., 2015; Luján et al., 2015; Frost et al., 2018).

The classic “hawk-dove” game describes a coexisting mechanism of higher animal species in sharing the same ecological niche. In the payoff matrix, the aggressive hawk can easily defeat the mild dove and gets the full resource V once encountered, while dove gets 0. When two hawks meet, each of them has a 50% chance to win or to loss the fight and will pay a cost C for fighting, thus the average outcome is V/2 – C/2. When two doves meet, they share the resource and get V/2. Because C is generally greater than V in the natural world, then V > V/2 > 0 > V/2 – C/2, thus coexistence of the two species is evolutionarily stable and the proportion of them depends on the probability of meeting (Maynard-Smith and Price, 1973; Auger et al., 1998). By contrast, accumulating theoretical and empirical evidence suggest that spatially structured environments can effectively promote the biodiversity of microbial communities by enhancing the local feedbacks to balance the proportions of individuals with different fitness (Turner and Chao, 1999; Kerr et al., 2002; Sachs et al., 2004; Ross-Gillespie et al., 2007; Santos et al., 2008; Pande et al., 2016). On the basis of these findings, it seems that the concept of hawk-dove game may also apply to microbial interspecific interaction, especially in spatially structured environments. Hence, we test this hypothesis by using the two Gram-negative pathogens *Pseudomonas aeruginosa* and *Klebsiella pneumoniae*, which naturally coexist in the gut of silk moth and can be coisolated from pneumonia and burn patients (Liu et al., 2004; Li et al., 2007; Anand et al., 2010; Kelvin Lee et al., 2016; Acosta et al., 2017), as experimental models.

The interspecific interaction and coexisting mechanisms of *P. aeruginosa* and *K. pneumoniae* in spatially structured communities were explored by coculturing them on solid media from different ratios. The distribution and proportion of the two species in mixed communities were determined by fluorescence imaging and CFU counting. A series of RNA-sequencing were further performed to pinpoint their transcriptional changes under different coculture conditions. The results showed that although *P. aeruginosa* generally had significant competitive advantage, the enhanced membrane-channel-related substance circulation of *K. pneumoniae* induced by the extracellular metabolites of *P. aeruginosa* enabled the invasion of *K. pneumoniae* toward *P. aeruginosa*. On the other side, when the two species were cocultured under the condition that *P. aeruginosa* had a metabolic burden, they could still invade each other’s population and coexist in the communities by forming a hawk-dove game like interactive relationship.

## Materials and Methods

### Bacterial Strains and Media

Strains of mCherry-labeled wild type *P. aeruginosa* PAO1 (WT PAO1), mCherry-labeled *P. aeruginosa* PAO1 isogenic *lasR* mutant (in-frame knockout) and green fluorescent protein (GFP)-labeled *K. pneumoniae* were previously preserved in the laboratory (Zhao et al., 2018). All the strains were routinely cultured in Luria-Bertani (LB) broth from a single colony.

### On-Plate Competition Experiments

Overnight cultured bacterial strains in LB broth were harvested and washed with a sterile saline solution (autoclaved 20 min at 127°C) for three times, and then adjusted to optical density at 600 nm (OD_600_) of 1.0. WT PAO1 or *lasR* mutant strains were well-mixed with *K. pneumoniae* at different ratios (1:99, 1:1, and 99:1) in 1.0 ml of sterile saline solution. Subsequently, 5 μl of inocula were separately spotted on disposable sterile petri dishes containing 20 ml of LB agar medium or modified M9 agar medium supplemented with 0.5% (w/v) of skim milk powder (Zhao et al., 2019). The plates were sealed by sealing films and then statically cultured at 37°C for different time periods. These experiments were independently repeated for six times. The growth status and distribution of bacterial cells were assessed by checking fluorescence using an IVIS XRII imaging system (Calpier, Inc.). Moreover, 1:1 mixed *P. aeruginosa* and *K. pneumoniae* were also stabbed (not reach the bottom) into LB plates containing 0.3% of agar using sterile toothpick or inoculated (2 μl of inocula) on the surface of LB plates containing 0.6% of agar to determine the swimming and swarming motilities of mixed communities, respectively. Pure cultures of *P. aeruginosa* and *K. pneumoniae* under the same conditions were set as controls.

### Competitivity Identification

At each sampling point of the competition experiments above, the colonies were gently scraped out from the plates by using autoclaved bamboo sticks with flat-shaped shovel at one end and dispersed in 1.0 ml of sterile saline solution, and then the bacterial cells were washed for three times. A fraction of appropriately diluted bacterial suspension was spread on M9-milk plate and cultured overnight (17 h) for species discrimination. WT PAO1 would form large colony size with clear proteolytic halo around the colony, *P. aeruginosa lasR* mutant would form relatively small colony without proteolytic halo, and *K. pneumonia* would form large colony size with no or faint proteolytic halo. The strains were further confirmed by IVIS XRII imaging to check the fluorescent colors or by colony PCR using specific primer pair 5′-CTTCATCGTCGGCAACTAC-3′ and 5′-GTCTGGTAGATGGACGGTTC-3′ to amplify the partial *lasR* gene of *P. aeruginosa*. Positive PCR-amplicon could only be obtained from WT PAO1. After calculating the proportion of each species in the mixed colonies at each sampling points, the relative fitness (*v*) of one species was then calculated by comparing the initial (x_0_) and final frequencies (x_1_) using the modified equation *v* = log10[x_1_ (1-x_0_)/x_0_ (1-x_1_)] based on previous description (Diggle et al., 2007a).

### Small Metabolites Mediated Interspecific Competition

Equal amounts (10 μl of bacterial suspension with OD_600_ of 1.0) of *P. aeruginosa* and *K. pneumoniae* were cocultured in disposable sterile 12-well polystyrene plate equipped with an inner well-containing a 0.4 μm permeable polycarbonate membrane (Corning, NY, United States) in the bottom of the inner well (Supplementary Figure 1). The two species were separated by the wall of the inner well, but could exchange the small extracellular metabolites through the membrane layer. The outer space of the well was supplied with 3.0 ml of LB broth and the inner space was supplied with 0.75 ml of LB broth. Coculture of 1:1 well-mixed *P. aeruginosa* and *K. pneumoniae* and pure cultures of the two species in normal 12-well polystyrene plate without membrane containing 4.0 ml of LB broth were set as control. All the capped plates were sealed by sealing films and statically cultured at 37°C for 3 days.

### RNA-Sequencing and Transcriptomic Analysis

Eight groups of experiments were carried out for RNA-sequencing (RNA-seq): (1) coculture (1:1) of *P. aeruginosa* and *K. pneumoniae* on LB plates; (2) pure culture of *P. aeruginosa* on LB plates; (3) pure culture of *K. pneumoniae* on LB plates; (4) coculture (1:1) of *P. aeruginosa* and *K. pneumoniae* in conventional 12-well plates containing LB broth; (5) coculture of *P. aeruginosa* (inoculated in the outer space) and *K. pneumoniae* (inoculated in the inner space) in LB broth in the 12-well plate equipped with an inner well (Supplementary Figure 1); (6) coculture of *K. pneumoniae* (inoculated in the outer space) and *P. aeruginosa* (inoculated in the inner space) in LB broth in the 12-well plate equipped with an inner well; (7) pure culture of *P. aeruginosa* in conventional 12-well plates containing LB broth; (8) pure culture of *K. pneumoniae* in conventional 12-well plates containing LB broth. All the cultures were statically incubated at 37°C for 3 days, and bacterial cells of each culture were then harvested for total RNA isolation using TRIzol reagents (Invitrogen). All the experiments were independently repeated for three times. RNA samples from three parallel cultures of each experiment above were well-mixed and conducted for library construction and RNA-seq using prokaryotic strand-specific Illumina-based RNA-seq technology (Novogene Bioinformatics Technology, Co., Ltd., China). The generated sequencing data are deposited in the NCBI database under the Accession No. PRJNA504726.

The software Bowtie 2 (Langmead and Salzberg, 2012) was used to map the clean reads from coculture of *P. aeruginosa* and *K. pneumoniae* on LB plate (Experiment 1), pure culture of *P. aeruginosa* on LB plate (Experiment 2), coculture of *P. aeruginosa* and *K. pneumoniae* in LB broth (Experiment 4), *P. aeruginosa* cultured in the outer well (Experiment 5), and pure culture of *P. aeruginosa* in LB broth (Experiment 7) to the reference genome of *P. aeruginosa* ^1^, respectively. Similarly, the clean reads from coculture of *P. aeruginosa* and *K. pneumoniae* on LB plate (Experiment 1), pure culture of *K. pneumoniae* on LB plate (Experiment 3), coculture of *P. aeruginosa* and *K. pneumoniae* in LB broth (Experiment 4), *K. pneumoniae* cultured in the outer well (Experiment 6), and pure culture of *K. pneumoniae* in LB broth (Experiment 8) to the reference genome of *K. pneumoniae*^2^, respectively. The software package Cufflinks (Trapnell et al., 2012) and DEGseq (Wang et al., 2010) were conducted to get transcriptome assembly and calculate the values of gene expression using expected fragments per kilobase of transcript per million fragments (FPKM). Differential gene expression was determined by comparing the FPKM values to those of monocultured corresponding species under the same growth condition (on plate or in liquid), respectively. For example, comparison of Experiment 1 to Experiment 2 indicates the profiling of differentially expressed genes of *P. aeruginosa* when *P. aeruginosa* was cocultured with *K. pneumoniae* on LB plate. The significance (*P*-value) of differentially expressed gene was corrected by calculating the false discovery rate *Q*, and *Q* < 0.05 was thought to be significantly different. Kyoto Encyclopedia of Genes and Genomes (KEGG) pathway prediction and protein classification analysis were performed by using KOBAS v2.0 (Mao et al., 2005).

### Statistical Analyses

Data analysis and statistical tests were performed by using the software GraphPad Prism version 7.0 (San Diego, CA, United States). Mean values of standard deviation were compared by using two-tailed unpaired *t*-test.

## Results and Discussion

### *P. aeruginosa* and *K. pneumoniae* Can Coexist in Structured Communities

**FIGURE 1:**
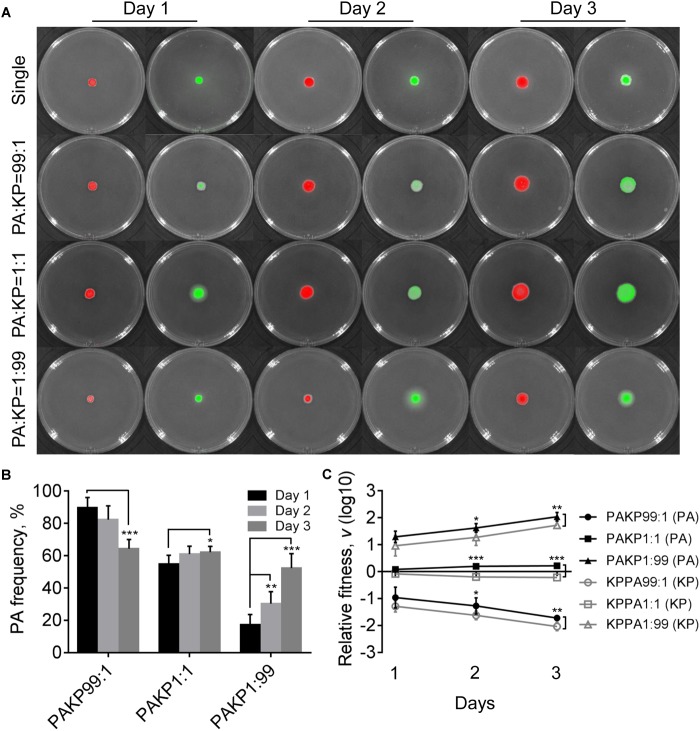
Competition of *Pseudomonas aeruginosa* (PA) and *Klebsiella pneumoniae* (KP) on LB plates. **(A)** Growth status and fluorescence detection of singly cultured *P. aeruginosa* or *K. pneumoniae* (first row) or coculture of them from different initial ratios. Red color, *P. aeruginosa*. Green color, *K. pneumoniae*. **(B)** Frequency of *P. aeruginosa* when cocultured with *K. pneumoniae* for different time periods. **(C)** Relative fitness of *P. aeruginosa* or *K. pneumoniae* when they were cocultured from different ratios. Data shown are mean values of standard deviation (*n* = 6). ^∗^*P* < 0.05, ^∗∗^*P* < 0.01, ^∗∗∗^*P* < 0.001. Two-tailed unpaired *t*-test.

To gain a general insight on the interactive relationship of *P. aeruginosa* and *K. pneumoniae* during static growth, we first grew the two species in different combinations on LB plates to monitor their frequencies. LB is a rich medium containing abundant resources which enables the normal growth of *P. aeruginosa* and *K. pneumoniae*. Our prior work showed that no significant growth difference was detected between monocultured *P. aeruginosa* and *K. pneumoniae* on LB plates, while *P. aeruginosa* had a competitive advantage over *K. pneumoniae* under coculture condition (Zhao et al., 2018). In this study, we found that in comparison to the moderately increased proportion of *P. aeruginosa* in the coculture with *K. pneumoniae* from an initial ratio of 1:1, either *P. aeruginosa* or *K. pneumoniae* from low initial proportion (1%) in the mixture could rapidly invade each other’s population (Figure 1A,B). In addition, the relative fitness of *P. aeruginosa* was always higher than *K. pneumoniae* under the same starting conditions after 2 days (Figure 1C), indicating that the invasion of *P. aeruginosa* toward *K. pneumoniae* was easier than the invasion of *K. pneumoniae* toward *P. aeruginosa* when they were cocultured on LB plate. Therefore, the unexpected mutual invasion relationship between *P. aeruginosa* and *K. pneumoniae* was similar with the outcome of hawk-dove game. This result was different from our previous finding that *P. aeruginosa* could overcome cocultured *K. pneumoniae*, while *K. pneumoniae* failed to invade *P. aeruginosa* in liquid culture with shaking (Zhao et al., 2018). We reasoned that this divergence could be due to the spatial distribution difference of bacterial cells between shaking and static culture conditions, since the structured population frequently led to coexistence of individuals with different relative fitness (Kerr et al., 2002; Ross-Gillespie et al., 2007; Santos et al., 2008; Pande et al., 2016).

Compared with the uniformly distributed *P. aeruginosa* in the communities, *K. pneumoniae* was excluded in the middle parts of colonies that grew from 99:1 or 1:1 mixture of *P. aeruginosa* and *K. pneumoniae* for 4 days (Supplementary Figure 2). As we had reported previously (Zhao et al., 2018), the peripheral distribution of *K. pneumoniae* was caused by the H1-T6SS (type VI secretion system) elicited cytotoxicity of *P. aeruginosa*, which could kill *K. pneumoniae* after 4 days of coculture. However, no inhibitory effect was observed when *P. aeruginosa* was cocultured with *K. pneumoniae* from an initial ratio of 1:99 (Supplementary Figure 2). This result indicated that in addition to the direct cell–cell contact with competitor cells as a necessary condition (Hood et al., 2010), high cell-density of *P. aeruginosa* might be a sufficient condition for the activation of *P. aeruginosa* T6SS during further interspecific competition. Moreover, we also noticed that *P. aeruginosa* had intrinsically stronger swimming ability than *K. pneumoniae*, and the mixed communities composed of both species showed remarkably enhanced swimming motility, instead of swarming, compared to the pure cultures (Supplementary Figure 3). Our previous work also found that the flagellum-encoding genes of *P. aeruginosa* were up-regulated during the coevolution with *K. pneumoniae* in liquid medium (Zhao et al., 2018). Therefore, these findings indicated that the presence of *K. pneumoniae* in the communities might improve the motility of *P. aeruginosa*.

### Enhanced Substance Circulation May Contribute to the Resistance of *K. pneumoniae* Against *P. aeruginosa* Suppression

We were interested to explore why *P. aeruginosa* and *K. pneumoniae* could invade each other by detecting the transcriptional changes of the two species under coculture condition using RNA-seq. To prevent the potential interference of T6SS-related cell toxicity, the colonies of mixed (1:1) and single species cultured on LB plates for 3 days were harvested for RNA-seq (Figure 1A). The transcriptional patterns of pure *P. aeruginosa* and *K. pneumoniae* were set as controls to determine the transcriptional changes of the two species under coculture condition.

**FIGURE 2:**
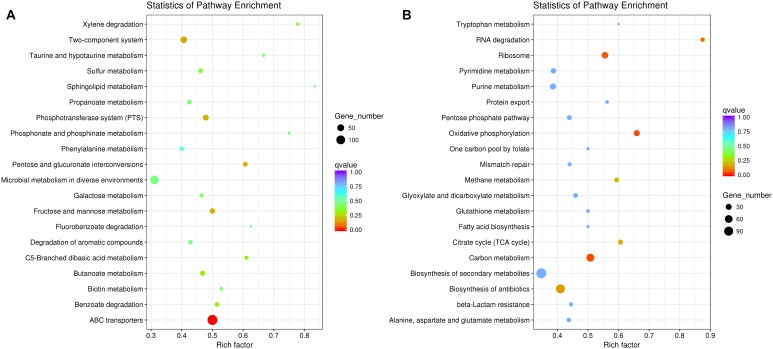
Significantly changed metabolic pathways of *K. pneumoniae* in the coculture with *P. aeruginosa* on LB plate compared to monoculture. **(A)** Up-regulated and **(B)** down-regulated pathways of *K. pneumoniae* when cocultured with *P. aeruginosa* for 3 days from an initial ratio of 1:1. Rich factor indicates the ratio of enriched gene number to the total annotated gene number in the pathway.

The result of comparative-transcriptomic analysis revealed that only eight significantly up-regulated genes and 147 down-regulated genes with no specific enrichment in any pathways were detected in *P. aeruginosa* when cocultured with *K. pneumoniae* (Supplementary Dataset 1). By contrast, a large scale of transcriptional change including 1734 significantly up-regulated genes enriched in ABC transporters (137 out of 274 genes, *P* = 9.10E-08) and 1116 down-regulated genes enriched in carbon metabolism (64 out of 126 genes, *P* = 0.034) and oxidative phosphorylation (31 out of 47 genes, *P* = 0.034) were detected in *K. pneumoniae* (Figure 2 and Supplementary Dataset 2). Additionally, when *P. aeruginosa* and *K. pneumoniae* were statically cocultured in LB broth, 55 significantly up-regulated genes enriched in aminobenzoate degradation (anthranilate biosynthesis, 3 out of 7 genes, *P* = 0.048) and 6 down-regulated genes with no specific enriched pathways were detected in *P. aeruginosa* (Supplementary Dataset 3). Similarly, 1334 significantly up-regulated genes enriched in ABC transporters (119 out of 274 genes, *P* = 3.01E-08) and benzoate degradation (20 out of 33 genes, *P* = 0.021) and 1220 down-regulated genes enriched in ribosome (46 out of 72 genes, *P* = 0.012), oxidative phosphorylation (33 out of 47 genes, *P* = 0.013), and carbon metabolism (66 out of 126 genes, *P* = 0.013) were detected in *K. pneumoniae* (Supplementary Dataset 4). These data suggested that *K. pneumoniae* was more susceptible to the presence of *P. aeruginosa*, and the enhanced membrane-channel-related substance circulation might contribute to the proportion increase of *K. pneumoniae* in the coculture with *P. aeruginosa*.

### Extracellular Metabolites Dominate the Competition Between *P. aeruginosa* and *K. pneumoniae*

**FIGURE 3:**
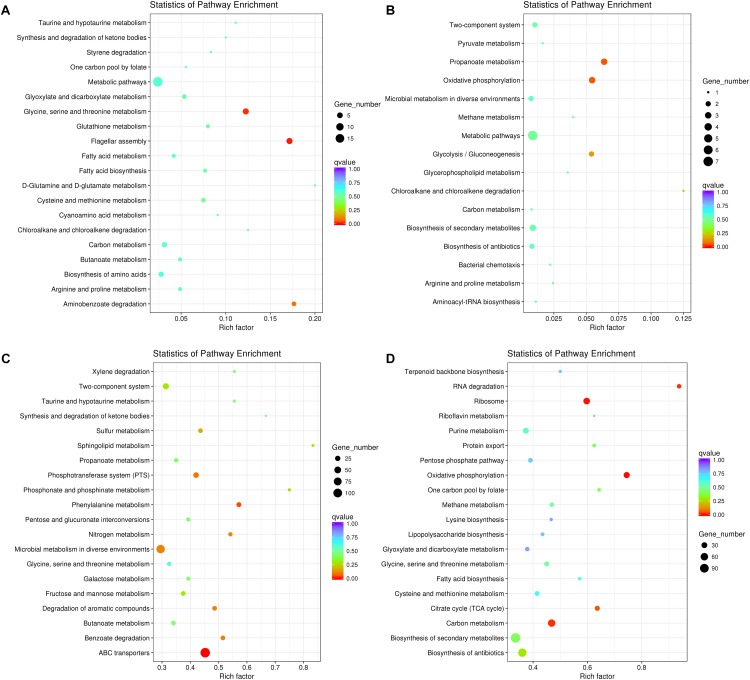
Significantly changed metabolic pathways of *P. aeruginosa* and *K. pneumoniae* induced by the extracellular metabolites of each other. **(A)** Up-regulated and **(B)** down-regulated pathways of *P. aeruginosa* cultured in the outer well for 3 days. *K. pneumoniae* was cocultured in the inner well. **(C)** Up-regulated and **(D)** down-regulated pathways of *K. pneumoniae* cultured in the outer well for 3 days. *P. aeruginosa* was cocultured in the inner well. See also Supplementary Figure 1 for more details of the equipment.

We further tested the possibility of extracellular-factor-mediated interspecific interaction between *P. aeruginosa* and *K. pneumoniae* by coculturing them in 12-well polystyrene plate containing a 0.4 μm permeable polycarbonate membrane (Supplementary Figure 1). In this way, growth of bacterial cells in outer well might be influenced by the secreted small metabolites from the species of inner well. When *P. aeruginosa* was cultured in outer well, the result of RNA-seq showed that 118 significantly up-regulated genes enriched in flagellar assembly (6 out of 35 genes, *P* = 0.005) and glycine, serine and threonine metabolism (6 out of 49 genes, *P* = 0.014), and 29 down-regulated genes with no specific enriched pathways were detected compared to monocultured *P. aeruginosa* (Figure 3A,B and Supplementary Dataset 5). The increased expression of flagellum-encoding genes under this condition further demonstrated that the swimming motility of *P. aeruginosa* might be enhanced by the stimulation of unknown extracellular metabolites from *K. pneumoniae*. Additionally, the expression of the pyoverdine biosynthesis operon, anthranilate biosynthesis operon and stress response related genes (such as *htpG*, *sigX*, *ibpA*, *groES*, and *hslV*) of *P. aeruginosa* were also significantly increased. These transcriptional changes were accordant to previous findings that, iron-competition was more frequent among interspecific bacterial species, and the expression of iron-chelators pyoverdine and *Pseudomonas* quinolone signal (PQS, the product of anthranilate metabolic pathway) were sensitive to the level of environmental iron. Moreover, PQS was capable of modulating various phenotypes including cell motility, biofilm production, and virulence, and could also influence the growth of other bacterial competitors such as *Staphylococcus aureus* and *Escherichia coli* (Mashburn et al., 2005; Diggle et al., 2007b; Tashiro et al., 2013; Khare and Tavazoie, 2015).

By contrast, when *K. pneumoniae* was cultured in outer well, 1379 significantly up-regulated genes enriched in ABC transporters (124 out of 274 genes, *P* = 1.60E-09) and phenylalanine metabolism (20 out of 35 genes, *P* = 0.038), and 1032 down-regulated genes enriched in oxidative phosphorylation (35 out of 47 genes, *P* = 0.001), ribosome (43 out of 72 genes, *P* = 0.003), carbon metabolism (59 out of 126 genes, *P* = 0.016), RNA degradation (15 out of 16 genes, *P* = 0.018), and citrate cycle (21 out of 33 genes, *P* = 0.047) were detected compared to monocultured *K. pneumoniae* (Figure 3C,D and Supplementary Dataset 6). These results combined with the up-regulated anthranilate biosynthesis operon of *P. aeruginosa* (Supplementary Dataset 5), indicating that the PQS of *P. aeruginosa* might suppress the growth and oxygen consumption rate of cultured *K. pneumoniae* by inhibiting the respiratory chain, like those happened during the interaction of *P. aeruginosa* with *E. coli* or *P. putida* (Toyofuku et al., 2010).

**Table 1:** Summary of significantly changed metabolic pathways of *P. aeruginosa* and *K. pneumoniae* under different coculture conditions compared to corresponding single culture (*Q* < 0.05).

	*P. aeruginosa*		*K. pneumoniae*	
	Up	Down	Up	Down
Solid medium	None	None	ABC transporters	Carbon metabolism
				Oxidative phosphorylation
Liquid medium	Aminobenzoate degradation	None	ABC transporters	Carbon metabolism
			Benzoate degradation	Oxidative phosphorylation
				Ribosome
Outer	Flagellar assembly	None	ABC transporters	Carbon metabolism
	Glycine, serine, and threonine metabolism		Phenylalanine metabolism	Oxidative phosphorylation
				Ribosome
				RNA degradation
				Citrate cycle

Finally, as summarized in Table 1, the small metabolites-directed transcriptional changes of *P. aeruginosa* and *K. pneumoniae* generally covered those cocultured on solid medium and in liquid condition. This result revealed that the interspecific competition between *P. aeruginosa* and *K. pneumoniae* were mainly dominated by extracellular products, and also confirmed the competitive advantage of *P. aeruginosa* and the role of *K. pneumoniae* membrane-transport systems in resisting the survival pressure imposed by *P. aeruginosa* (Khare and Tavazoie, 2015). Taken together, although the mutual invasion relationship of *P. aeruginosa* and *K. pneumoniae* identified in the current study was similar to the hawk-dove game, their interior interacting dynamics might be different. Unlike the intense intraspecific competition of hawk and its absolute advantage over dove, the invasion of *P. aeruginosa* and *K. pneumoniae* toward each other should pay additional cost in producing specific factors. Moreover, the enhanced substance circulation of *K. pneumoniae* identified in our current study might also provide a plausible explanation for the increased tolerance of mixed population against sodium dodecylsulfate stress (Kelvin Lee et al., 2016). Further more detailed study concerning the roles of toxic factors and membrane transporters would significantly contribute to understanding the formation and stabilization mechanisms of polymicrobial communities in diverse environments.

### Extracellular Products Mediated Coexistence of *P. aeruginosa* and *K. pneumoniae*

To investigate the extracellular product-based interspecific interaction between *P. aeruginosa* and *K. pneumoniae*, another batch of on-plate competition assay was performed by using M9 medium containing sodium caseinate or skim milk powder as the sole carbon source. Previous studies reported that *P. aeruginosa* can grow in M9-casein (or milk) by synthesizing quorum-sensing (QS) system-controlled costly extracellular product elastase (Wilder et al., 2011; Darch et al., 2012). *K. pneumoniae* is also capable of producing this proteolytic enzyme, albeit its encoding gene still remained unknown (Trishin et al., 2004, 2005). Indeed, both *P. aeruginosa* and *K. pneumoniae* can grow on M9-milk plates, and the growth rate of *P. aeruginosa* was significantly slower than *K. pneumoniae* (Supplementary Figure 4). However, compared with the apparent proteolytic ring formed around *P. aeruginosa* colony, there was only a faint halo around *K. pneumoniae* colony. These results combined with the equal growth rates of *P. aeruginosa* and *K. pneumoniae* on rich medium (Zhao et al., 2018), indicating that the production of elastase through energy-intensive QS-regulation might burden the growth of *P. aeruginosa* on M9-milk plate.

**FIGURE 4:**
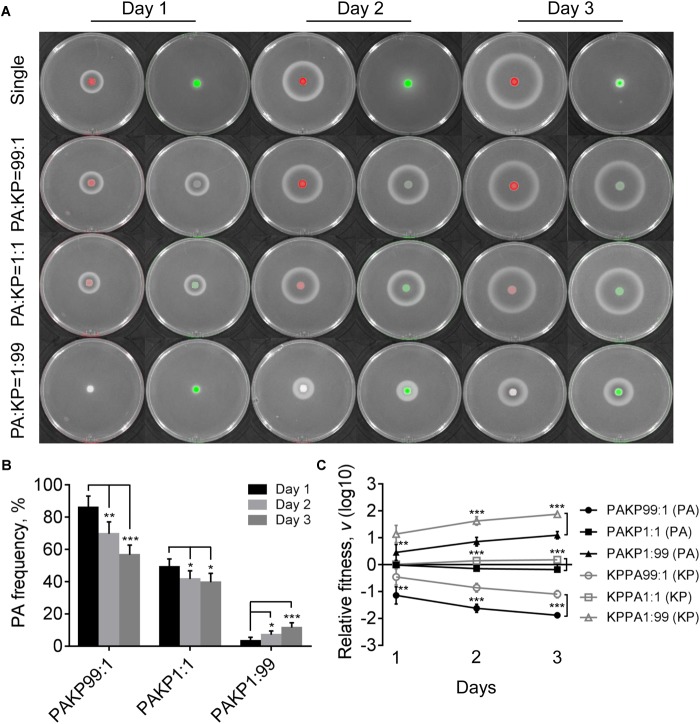
Competition of *P. aeruginosa* and *K. pneumoniae* on M9-milk plates. **(A)** Growth status and fluorescence detection of singly cultured *P. aeruginosa* or *K. pneumoniae* (first row) or coculture of them from different initial ratios. Red color, *P. aeruginosa*. Green color, *K. pneumoniae*. **(B)** Frequency of *P. aeruginosa* when cocultured with *K. pneumoniae* for different time periods. **(C)** Relative fitness of *P. aeruginosa* or *K. pneumoniae* when they were cocultured from different ratios. Data shown are mean values of standard deviation (*n* = 6). ^∗^*P* < 0.05, ^∗∗^*P* < 0.01, ^∗∗∗^*P* < 0.001. Two-tailed unpaired *t*-test.

In contrast to the competitive advantage of *P. aeruginosa* over *K. pneumoniae* on LB plates (Figure 1), the proportion of *P. aeruginosa* decreased gradually when cocultured with *K. pneumoniae* on M9-milk plate from an initial ratio of 1:1, while either *P. aeruginosa* or *K. pneumoniae* from low proportion (1%) in the mixture could still invade each other (Figure 4A,B). The results of relative fitness analyses further revealed that *K. pneumoniae* always grew faster than *P. aeruginosa* when they were cocultured on M9-milk plates from any initial ratios (Figure 4C). These findings suggested that *K. pneumoniae* had a competitive advantage over *P. aeruginosa* when cocultured on M9-milk plates, and the invasion of *K. pneumoniae* toward *P. aeruginosa* was easier than the invasion of *P. aeruginosa* toward *K. pneumoniae*. Additionally, the proportion increase of *P. aeruginosa* cultured from low proportion (1%) on M9-milk plates was significantly delayed compared with that on LB plates, and also significantly slower than *K. pneumoniae* under the same given condition (Supplementary Figure 5). Intriguingly, *K. pneumoniae* also emerged in the region of proteolytic ring formed by *P. aeruginosa* (Supplementary Figure 6), indicating that *K. pneumoniae* might benefit from the extracellular products of *P. aeruginosa*. Therefore, our finding here revealed that although the development of QS system endowed *P. aeruginosa* with an advantage in resource competition (Zhao et al., 2018), *K. pneumoniae* was capable of overcoming *P. aeruginosa* depending on the low metabolic cost or by stealing the extracellular elastase produced by *P. aeruginosa*. Furthermore, no T6SS-related inhibitory effect of *P. aeruginosa* on *K. pneumoniae* like that on LB plate was observed even when they were cocultured for 4 days (Supplementary Figure 7). This could be explained by the finding that the activation of H1-T6SS was negatively regulated by the central QS regulator LasR (Lesic et al., 2009), and thus the H1-T6SS-mediated killing of *P. aeruginosa* on *K. pneumoniae* would not happen under the condition requiring an active LasR regulon for growth.

### *P. aeruginosa* QS-Deficiency Contributes to the Coexistence With *K. pneumoniae*

**FIGURE 5:**
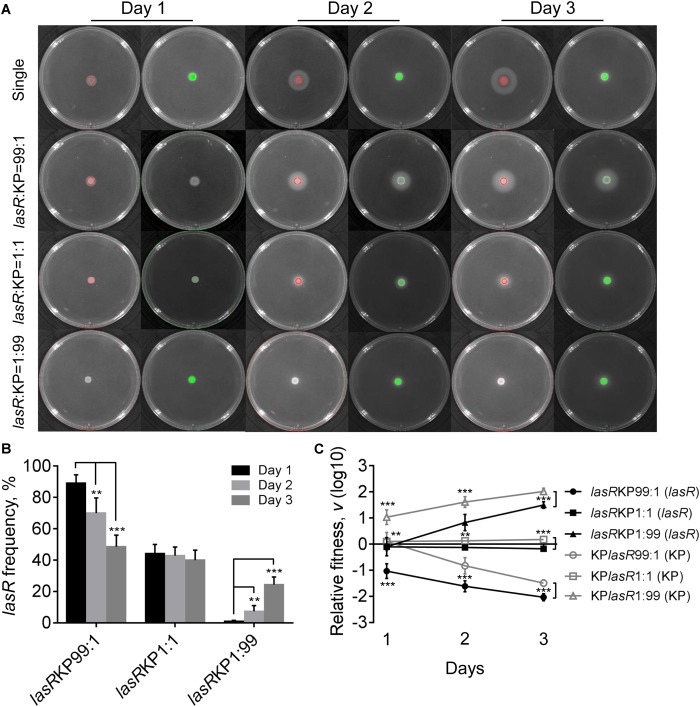
Competition of *P. aeruginosa lasR* mutant (*lasR*) and *K. pneumoniae* (KP) on M9-milk plates. **(A)** Growth status and fluorescence detection of singly cultured *P. aeruginosa lasR* mutant or *K. pneumoniae* (first row) or coculture of them from different initial ratios. Red color, *P. aeruginosa lasR* mutant. Green color, *K. pneumoniae*. **(B)** Frequency of *P. aeruginosa lasR* mutant when cocultured with *K. pneumoniae* for different time periods. **(C)** Relative fitness of *P. aeruginosa lasR* mutant or *K. pneumoniae* when they were cocultured from different ratios. Data shown are mean values of standard deviation (*n* = 6). ^∗^*P* < 0.05, ^∗∗^*P* < 0.01, ^∗∗∗^*P* < 0.001. Two-tailed unpaired *t*-test.

Evolution of *P. aeruginosa* under QS-required condition *in vitro* or in cystic fibrosis lungs frequently selects *lasR* mutants with impaired QS-regulation to reduce the cost in synthesizing extracellular factors (Sandoz et al., 2007; Köhler et al., 2009; Smith and Chapman, 2010; Zhao et al., 2016). To further investigate the role of *P. aeruginosa* QS system in extracellular product-mediated competition with *K. pneumoniae*, the *lasR* mutant strain of *P. aeruginosa* with deficient elastase production ability was used to repeat the competition assay with *K. pneumoniae* on M9-milk plates. *P. aeruginosa lasR* mutant had been defined as a typical cheater which could invade QS-intact cooperators by stealing the extracellular products in the population (Sandoz et al., 2007; Wilder et al., 2011). We found that although the growth of *P. aeruginosa lasR* mutants at high cell-densities was poor, they could also produce less elastase during prolonged culture through an uncharacterized RhlR-dependent signaling (Figure 5A, Dandekar and Greenberg, 2013).

The results of coculture assays showed that either *P. aeruginosa lasR* mutant or *K. pneumoniae* could readily invade each other’s population, and no significant proportion changes and relative fitness of *P. aeruginosa lasR* mutant and *K. pneumoniae* were detected when they were cocultured from an initial ration of 1:1 (Figure 5 and Supplementary Figure 8). Moreover, the proportion increase of *lasR* mutant was significantly faster than WT PAO1 when competing with *K. pneumoniae* from a low initial proportion of 1% for 3 days (Supplementary Figure 9). Therefore, our data here suggested that when the QS-mediated metabolic burden or competitive advantage of *P. aeruginosa* was removed, *K. pneumoniae* could invade *P. aeruginosa* by the innate growth advantage on M9-milk medium, and *P. aeruginosa lasR* mutant had the ability to invade *K. pneumoniae* in turn by reaping the limited elastase produced by *K. pneumoniae*. Collectively, although the competitive advantages of *P. aeruginosa* and *K. pneumonias* were alterable in dependence of population structure and nutrient conditions, mutual cheating and metabolic advantage might jointly contribute to the hawk-dove game like coexisting of *P. aeruginosa* and *K. pneumoniae* in spatially structured communities.

## Conclusion

This study provides evidence that *P. aeruginosa* and *K. pneumoniae* have the capacities to invade each other and can coexist in structured communities by forming a hawk-dove game like interactive relationship. These findings highlight the importance of spatially structured environments in the formation and stabilization of polymicrobial communities, open an avenue for further understanding the complex interspecific interactions of microbes during coevolution, and may also have general implications on the development of social microbiology and novel clinical therapies.

## Author Contributions

KZ designed the study and wrote the manuscript. KZ, JL, TH, YY, and JfL performed the experiments. KZ and BY coordinated the bioinformatics analysis. XW and YC coordinated the laboratory management and materials collection.

## Conflict of Interest Statement

The authors declare that the research was conducted in the absence of any commercial or financial relationships that could be construed as a potential conflict of interest.

## Footnotes

1 www.ncbi.nlm.nih.gov/nuccore/AE004091.2

2 https://www.ncbi.nlm.nih.gov/nuccore/NZ_CP014696
