# Supplementary material for: Coexistence of Microbial Species in Structured Communities by Forming a Hawk-Dove Game Like Interactive Relationship
Source: Front Microbiol. 2019 Apr 16;10:807. doi: 10.3389/fmicb.2019.00807 (PMC6477097; doi:10.3389/fmicb.2019.00807)
Supplement: Supplementary file 7 [file Data_Sheet_1.PDF]

Supplementary Material

Coexistence of Microbial Species in Structured Communities by Forming a Hawk-dove Game Like Interactive Relationship

Kelei Zhao[1,*], Jing Li [1], Ting Huang[1], Yang Yuan[1], Jiafu Lin[1], Bisong Yue[2], Xinrong Wang[1], and Yiwen Chu[1,*]

[1] Antibiotics Research and Re-evaluation Key Laboratory of Sichuan Province, Sichuan Industrial Institute of Antibiotics, Chengdu University, Chengdu, China

[2] Key Laboratory of Bio-resources and Eco-environment (Ministry of Education), College of Life Sciences, Sichuan University, Chengdu, China

*** Correspondence:**
Kelei Zhao, Email: zkl5228@163.com; and Yiwen Chu, Email: chuyiwen@cdu.edu.cn.

Supplementary Figures

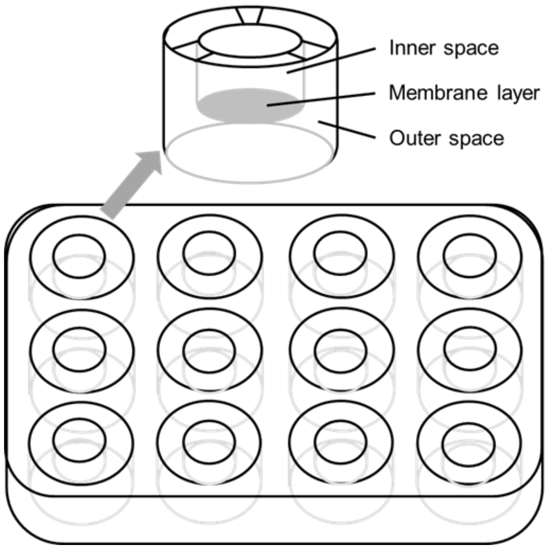

Supplementary Figure 1. Sketch of 12-well polystyrene plate equipped with an inner well containing a 0.4 μm permeable polycarbonate membrane in the bottom of the inner well.

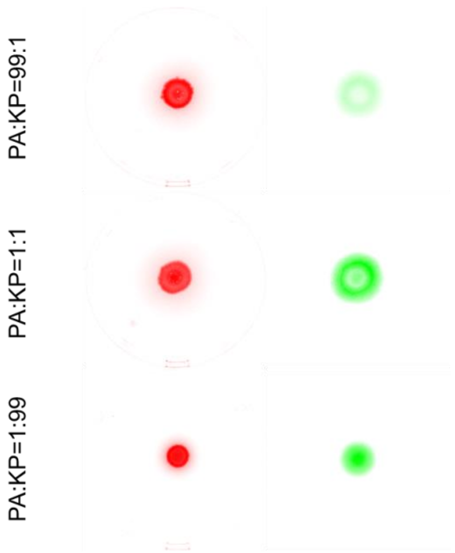

Supplementary Figure 2. Fluorescence detection of cocultured *P. aeruginosa* and *K. pneumoniae* from different initial ratios on LB plates for 4 days. Red color, *P. aeruginosa*. Green color, *K. pneumoniae*.

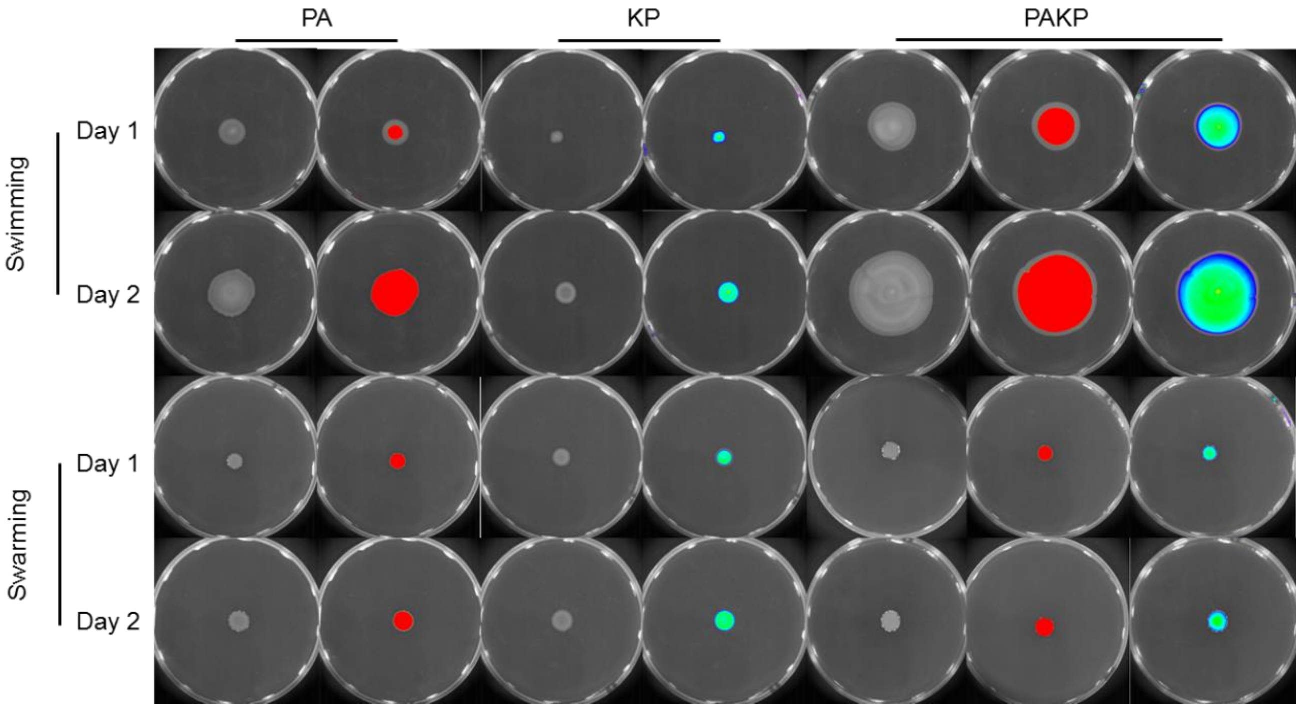

Supplementary Figure 3. Motilities of *P. aeruginosa* (PA) and *K. pneumoniae* (KP) on under mono- or co-culture conditions. Swimming abilities of PA, KP, or 1:1 mixture of them (PAKP) were determined by stabbing (not reach the bottom) the inocula into LB plates containing 0.3% of agar. Swarming abilities were determined by inoculating 2 μl of inocula on the surface of LB plates containing 0.6% of agar. Growth status of PA and KP were measured after 1 and 2 days by fluorescence detection. Red color, *P. aeruginosa*. Green color, *K. pneumoniae*.

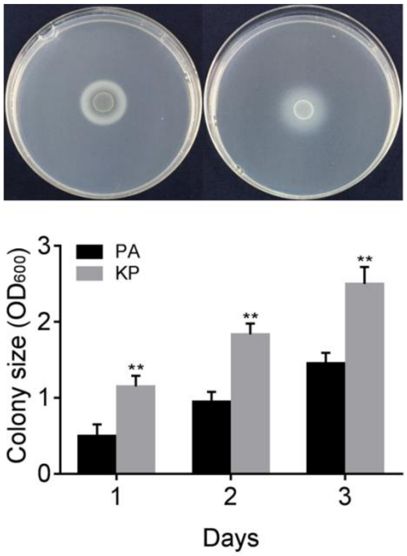

Supplementary Figure 4. Growth status of *P. aeruginosa* (PA) and *K. pneumoniae* (KP) on M9-milk plates. Upper left, *P. aeruginosa*. Upper right, *K. pneumoniae*. Mean values of standard deviation (n = 3) of each column were compared to *P. aeruginosa* at each time-point by using two-tailed unpaired *t*-test. **, $P < 0.01$.

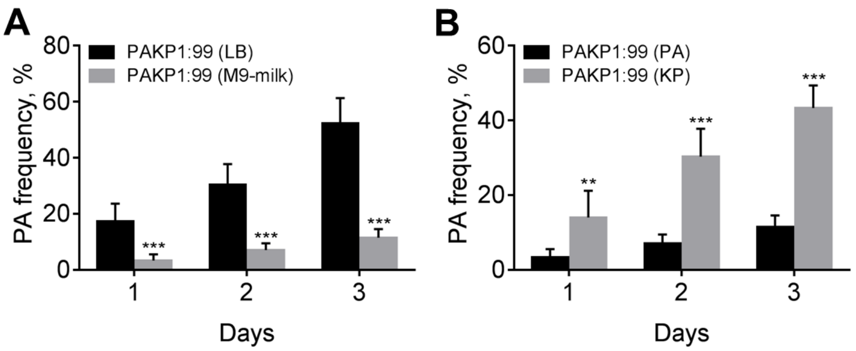

Supplementary Figure 5. (A) Proportional changes of *P. aeruginosa* cocultured with *K. pneumoniae* from an initial ratio of 1:99 on different solid media. Mean values of standard deviation (n = 6) of each column were compared to LB plates at each time-point by using two-tailed unpaired t-test. ***, $P < 0.001$. **(B)** Proportional changes of *P. aeruginosa* or *K. pneumoniae* cocultured from an initial ratio of 1:99 with each other on M9-milk plates. Mean values of standard deviation (n = 6) of each column were compared to *P. aeruginosa* at each time-point by using two-tailed unpaired t-test. **, $P < 0.01$, ***, $P < 0.001$.

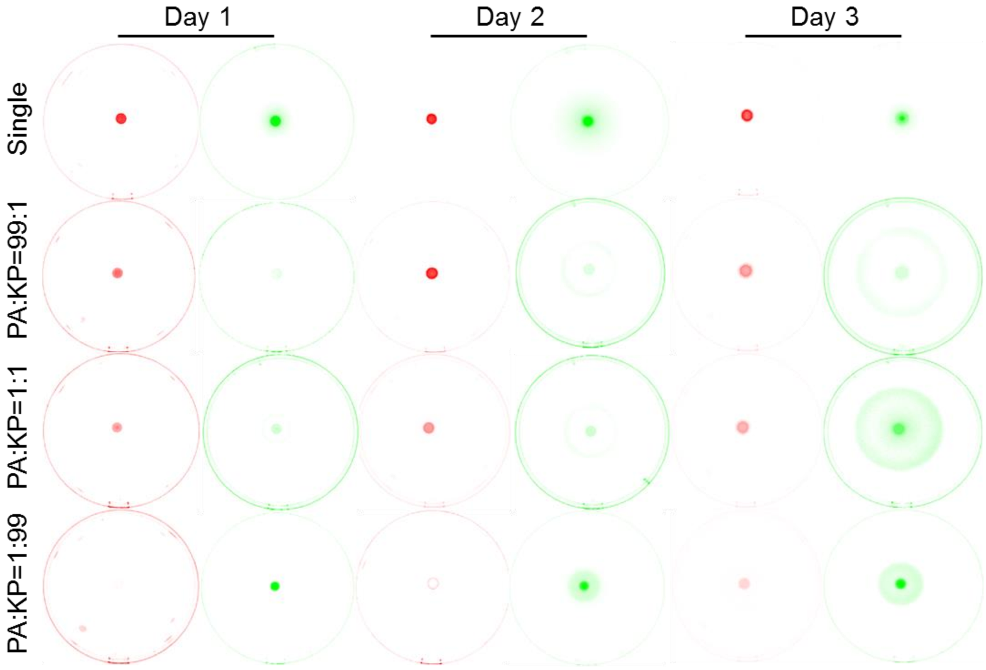

Supplementary Figure 6. Fluorescence detection of singly cultured *P. aeruginosa* or *K. pneumoniae* (first row) or coculture of them from different initial ratios on M9-milk plates. Red color, *P. aeruginosa*. Green color, *K. pneumoniae*.

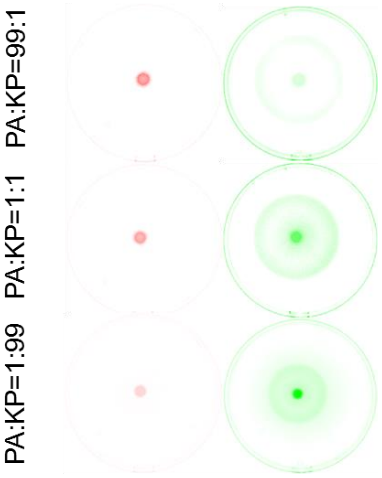

Supplementary Figure 7. Fluorescence detection of *P. aeruginosa* and *K. pneumoniae* cocultured from initial ratio of 1:1 on M9-milk plates for 4 days. Red color, *P. aeruginosa*. Green color, *K. pneumoniae*.

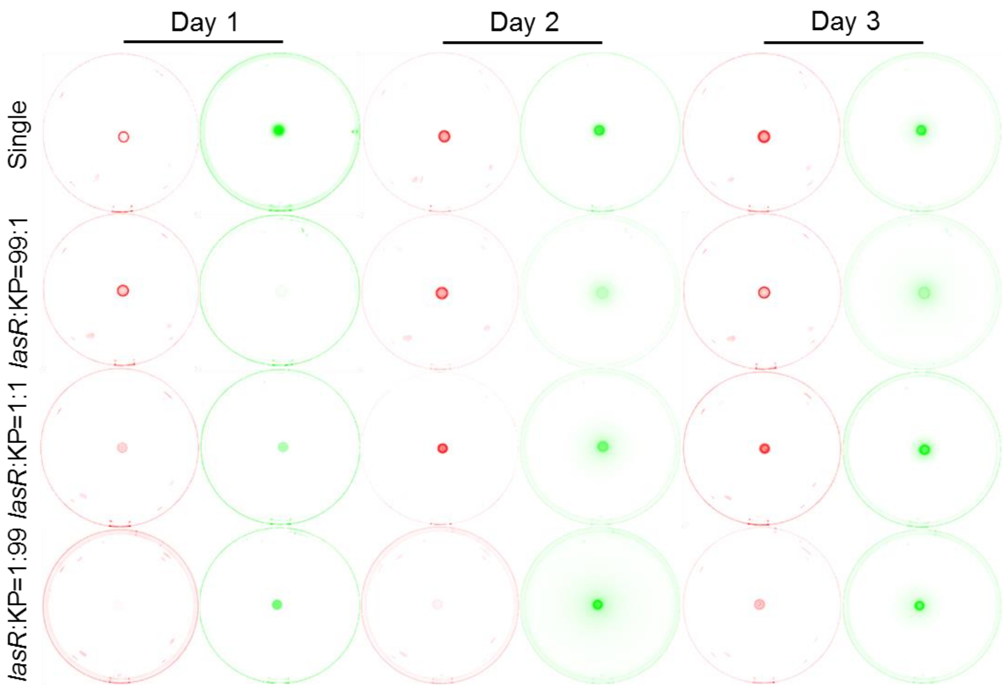

Supplementary Figure 8. Fluorescence detection of singly cultured *P. aeruginosa lasR* mutant (*lasR*) or *K. pneumoniae* (first row) or coculture of them from different initial ratios on M9-milk plates. Red color, *P. aeruginosa lasR* mutant. Green color, *K. pneumoniae*.

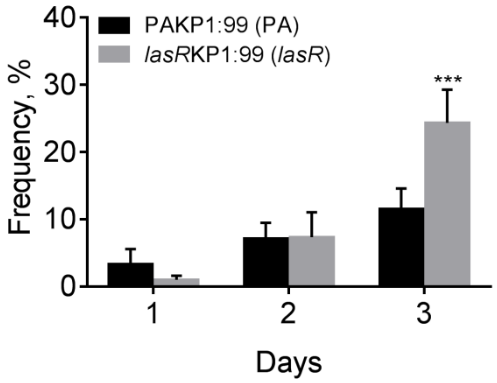

Supplementary Figure 9. Proportional changes of *P. aeruginosa* or *P. aeruginosa lasR* mutant cocultured with *K. pneumoniae* from an initial ratio of 1:99 on M9-milk plates. Mean values of standard deviation (n = 6) of each column were compared to *P. aeruginosa* at each time-point by using two-tailed unpaired *t*-test. ***, $P < 0.001$.

Supplementary Datasets

Supplementary Dataset 1. Significantly differentially expressed genes of *P. aeruginosa* when statically cocultured with *K. pneumoniae* on LB agar for 3 days.

Supplementary Dataset 2. Significantly differentially expressed genes of *K. pneumoniae* when statically cocultured with *P. aeruginosa* on LB agar for 3 days.

Supplementary Dataset 3. Significantly differentially expressed genes of *P. aeruginosa* when statically cocultured with *K. pneumoniae* in LB broth for 3 days.

Supplementary Dataset 4. Significantly differentially expressed genes of *K. pneumoniae* when statically cocultured with *P. aeruginosa* in LB broth for 3 days.

Supplementary Dataset 5. Significantly differentially expressed genes of *P. aeruginosa* cultured in the outer well compared to normal pure culture.

Supplementary Dataset 6. Significantly differentially expressed genes of *K. pneumoniae* cultured in the outer well compared to normal pure culture.
